# ViroMatch: A Computational Pipeline for the Detection of Viral Sequences from Complex Metagenomic Data

**DOI:** 10.1128/MRA.01468-20

**Published:** 2021-03-04

**Authors:** Todd N. Wylie, Kristine M. Wylie

**Affiliations:** a Department of Pediatrics, Washington University School of Medicine, St. Louis, Missouri, USA; Indiana University, Bloomington

## Abstract

ViroMatch is an automated pipeline that takes metagenomic sequencing reads as input and performs iterative nucleotide and translated nucleotide mapping to identify viral sequences. We provide a Docker image for ViroMatch, so that users will not have to install dependencies.

## ANNOUNCEMENT

Next-generation sequencing (NGS) is a powerful tool that allows the comprehensive characterization of viral communities and the discovery of novel viruses (1–5). While software pipelines exist for viral detection (6–8), many rely on prohibitive memory and CPU requirements; others rely on stringent *k*-mer hashing that lacks sensitivity. We developed ViroMatch to analyze data sets of millions of short metagenomic sequence reads to identify viruses by sensitive sequence alignment using pragmatic system resources. We have modified and refined our analysis pipeline workflow over time, primarily applied to the analysis of vertebrate viruses (9–16). The latest version is made available to the public for the first time here.

**FIG 1:**
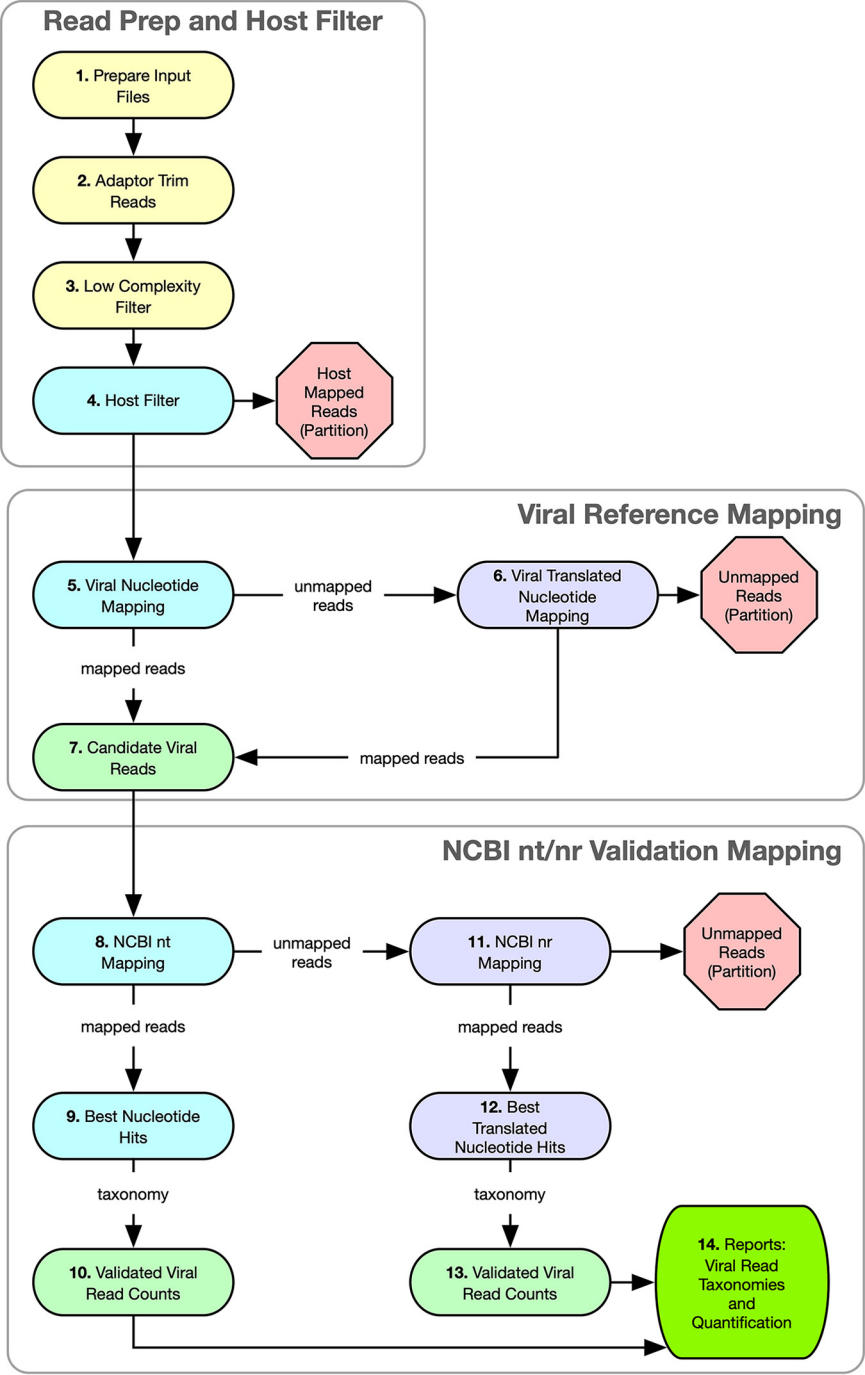
ViroMatch pipeline workflow. (1 to 3) Input sequences are prepared for processing. Sequencing adaptors are excised, low-quality base pairs are trimmed from read ends, and the resultant too-short reads are removed. Low-complexity and repetitive base pairs are soft masked. (4) The reads are mapped to the host reference genome. Reads that map to the host are partitioned for later referral. Nonhost reads are promoted for mapping to the virus-only database. (5 to 7) The reads are first mapped to a virus-only reference genome database using nucleotide alignment. Reads that did not map are subsequently mapped to a translated nucleotide version of the same virus-only database. Reads that did not map at all are partitioned for later referral. The mapped reads (both nucleotide and translated nucleotide) are collected for validation mapping. (8 to 13) Candidate viral reads are validated by mapping to comprehensive NCBI nt and nr references in an iterative approach similar to steps 5 to 7. The best hit for each read is chosen using a predefined algorithm that determines viral positivity and taxonomic classification. (14) Viral read counts and taxonomic classifications are compiled into reports.

The ViroMatch workflow is shown in Fig. 1. All reads are host filtered prior to viral assessment. Metagenomic sequences are screened for putative viral reads by nucleotide mapping (BWA-MEM [17]) and translated mapping (Diamond [18]) against a database of virus-specific reference genome sequences collected from NCBI GenBank (19). The use of both nucleotide and translated amino acid sequence alignments enables the detection of sequences that are conserved or divergent compared to reference genome sequences. This first screen is fast, but the hits include false positives; therefore, the putative viral hits are subsequently mapped to the more comprehensive NCBI nucleotide (nt) and NCBI nonredundant (nr) amino acid databases (https://www.ncbi.nlm.nih.gov/). Only sequences with an unambiguous mapping to a viral reference are counted as viral hits. Ambiguous hits—e.g., those mapping with similar scores to viruses, human, bacteria—are not counted. Ambiguous hits also include those that map to repetitive regions not suitable for determining virus positivity.

Upon completion, ViroMatch provides reports detailing viral taxonomic classification and quantification of mapped reads. Report read counts have been mapped to the virus-only reference database, have undergone validation of candidate viral reads against local NCBI nt and nr reference databases, have been taxonomically classified, and have been filtered by best-hit logic. Only reads that have passed all of these steps are considered viral identities.

The system requirements related to disk space and memory vary depending on the size of the samples being processed; however, we recommend a minimum of 16 Gb of memory. We have run ViroMatch on thousands of samples, ranging from 1 to 200 million reads processed per sample. As an example, ViroMatch processed ∼11 million reads using a single core and 16 Gb of RAM with a total runtime of 8:00:06. ViroMatch uses Snakemake (20) to transparently organize and run all of its steps.

### Data availability.

ViroMatch is available through an executable Docker (21) image (https://hub.docker.com/r/twylie/viromatch), which contains all necessary code and third-party dependencies. ViroMatch’s required reference genome databases are also available for download (https://twylie.github.io/viromatch/download_and_install/databases/) using Globus (22, 23) data transfer software. ViroMatch pipeline source code is also available through GitHub (https://github.com/twylie/viromatch) and is provided under the MIT license. For download and installation instructions and complete usage documentation, please visit the ViroMatch website at https://twylie.github.io/viromatch/.
